# “*I eat the* manofê *so it is not forgotten*”: local perceptions and consumption of native wild edible plants from seasonal dry forests in Brazil

**DOI:** 10.1186/1746-4269-10-45

**Published:** 2014-05-23

**Authors:** Margarita Paloma Cruz, Patrícia Muniz Medeiros, Iván Sarmiento Combariza, Nivaldo Peroni, Ulysses Paulino Albuquerque

**Affiliations:** 1 Laboratory of Applied and Theoretical Ethnobiology (LEA), Biology Department, Botany Area, Federal Rural University of Pernambuco, Av. Dom Manoel de Medeiros s/n, Dois Irmãos, CEP: 52171–900, Recife Pernambuco, Brazil; 2 Colombian Society of Ethnobiology, Bogotá, Colombia; 3 Institute of Biological Sciences and Sustainable Development, Federal University of Bahia, Bahia, Brazil; 4 Escuela de Medicina y Ciencias de la Salud, Universidad del Rosario, Bogotá, Colombia; 5 Centro de Estudios Médicos Interculturales (CEMI), Cota Cundinamarca, Colombia; 6 Ecology and Zoology Department (ECZ), Federal University of Santa Catarina, Santa Catarina, Brazil

**Keywords:** Ethnobotany, Ethnobiology, Local representations, Consumption preferences, Food plants

## Abstract

**Background:**

There is little information available on the factors influencing people’s selection of wild plants for consumption. Studies suggest a suitable method of understanding the selection of edible plants is to assess people’s perceptions of these resources. The use and knowledge of wild resources is disappearing, as is the opportunity to use them. This study analyzes people’s perceptions of native wild edible plants in a rural Caatinga (seasonal dry forest) community in Northeast Brazil and the relationships between the use of these resources and socioeconomic factors.

**Methods:**

Semi-structured interviews with 39 people were conducted to form a convenience sample to gather information regarding people’s perceptions of 12 native wild edible plant species. The relationships between variables were assessed by simple linear regression analysis, Pearson and Spearman correlation analyses, and in the case of nominal variables, contingency tables. The discourse of participants regarding their opinions of the use of wild plants as food was analyzed through the collective subject discourse analysis technique.

**Results:**

Perceptions were classified into 18 categories. The most cited category was *organoleptic characteristics of the edible part*; more specifically, flavor. Flavor was the main positive perception associated with plant use, whereas the negative perception that most limited the use of these plants was *cultural acceptance*. Perceptions of the use of wild edible plants were directly correlated with both interviewee age and income.

**Conclusion:**

Within the studied community, people’s perceptions of native wild edible plants are related to their consumption. Moreover, the study found that young people have less interest in these resources. These findings suggest that changing perceptions may affect the conservation of plants, traditional practices and the associated knowledge.

## Background

The use of edible plants is a universal human practice because it satisfies the most fundamental of basic needs [1], nutrition. Human beings have always explored their surroundings in search of nutritional resources, selecting attractive plants and ignoring others [2,3]. Several factors attract people to certain plants over others, thus limiting the range of plants used for food [2,4,5].

The study of factors that influence the selection of plants for specific purposes has become increasingly popular in ethnobotanical studies [6-10], and several hypotheses have been advanced. However, few studies have investigated the factors that influence the selection of wild edible plants, and even fewer that do so approach it from an ethnobotanical perspective.

Research in Latin America, Africa, and Asia has shown that the study of people’s perceptions of edible plants is an effective method for understanding selection patterns and subsequent plant use [11-13]. Therefore, the results of these studies reflect the reality of the studied communities.

Previous studies have addressed the physiological factors, such as the effects of flavor on selection of food and medicinal resources; however perception also comprises psychological and cultural aspects [14-17]. In addition, studies have addressed cognitive factors that affect the perception of plant resources. Some authors have stated that greater importance should be placed on local taxonomy when studying perception [15]. Others have argued that comparisons between similar human populations from similar environments are needed to better understand why certain species are consumed while others are not [18].

The present study proposes a framework similar to studies conducted in Argentina [11] and Sudan [12]. Ladio [11] studied Mapuches populations in Argentina and found that the main factors favoring the continued use of some wild edible plants were an interest in maintaining tradition, plant flavor, and a need to utilize alternative food sources.

Grosskinsky and Gullick [12] questioned members of a community in southern Sudan about their perceptions of wild edible plants. The authors found that the community considered the diversity that the plants provided to their diet and the additional economic income they provided to be advantages. In contrast, reported disadvantages were the long distances sometimes required to access the plants and the negative perception that members of the community had for some plants.

Reduced consumption and knowledge of edible wild plants is a threat to food security [19]. Moreover, the use and management of natural resources fosters environmental conservation [20].

Therefore, the present study aims to evaluate the perceptions of people from a rural *Caatinga* community regarding wild edible plants and to investigate the relationships between these perspectives, use of plants and socioeconomic factors. Two hypotheses are tested. First, we tested the hypothesis that the consumption of native wild edible plants correlates with people’s perceptions. Specifically, it is expected that a) the most consumed plants are those perceived as tasting better and those that are commercialized and b) the least consumed plants are those that are viewed negatively by the community and those that are not readily available. Second, we hypothesized that people’s perceptions of native wild edible plants are correlated with socioeconomic factors in the studied rural community. Specifically, we expected that a) people with lower incomes or who perform agricultural activities have perceptions that encourage the use of wild edible plants and b) people with higher incomes or who do not perform agricultural activities have perceptions that limit the use of wild edible plants. To test our hypotheses, we evaluated the relationships between perceptions and the use of both food items and socioeconomic factors. The indicators used to evaluate these relationships are shown in Table 1.

**Table 1:** Variables studied to analyze the relationships between perceptions, the use of native wild edible plants, and socioeconomic factors in a local community in NE Brazil

**Perceptions**	**Indicators of use and socioeconomic factors**	
Number of citations for each perceptions category	Use	Number of edible items currently used
		Number of edible items used in the past
	Socioeconomic factors	Age
		Gender
		Past occupation
		Current occupation
		Monthly family income
		Monthly individual income

### Study area

The study included residents of the rural Carão community (189 inhabitants, 112 over 18 years old) [21] in the municipality of Altinho (8° 29′23″ S and 36° 03′34″ W) in the central hinterland of the state of Pernambuco [22]. The community is 16 km from the center of Altinho, which is 160 km from Recife, the state capital.

Carão has a hot, semi-arid climate (469 m above sea level), an average temperature of 25°C, and an annual rainfall of approximately 622 mm that is concentrated between March and July [23]. *Caatinga* vegetation (seasonal dry forest) dominates the region [22].

The community is typically rural, and subsistence farming is the main activity. Farming is conducted mainly during the rainy season, and the main crops are monocultures of corn and beans, which are complemented by cattle and goat farming [24,25]. Products in excess of the families’ needs are sold in the weekly market in the center of Altinho, generating income for the community [25]. The scarcity of local jobs and the desire of youth to achieve higher income levels has led many young inhabitants to move to Recife or São Paulo in search of better job opportunities [25]. Therefore, the community is increasingly devoid of middle-aged people and is dominated by children and the elderly.

Residents of the Carão community consume wild plants opportunistically. The wild edible plants that occur near places of everyday activities are more frequently used as food resources [26]. According to Nascimento et al. [27], the habit of collecting and consuming such plants is being abandoned, partly because people prefer to consume cultivated species or foods that can be locally purchased.

## Methods

### Participant selection

Convenience sampling was performed among heads of household who had been interviewed previously by other members of the *Laboratory of Applied and Theoretical Ethnobiology* (Portuguese acronym: LEA) [21,24,25,27-31]. The mention of at least 3 edible species was used as the selection criterion. A total of 44 people were selected, 39 of whom completed the interview. The remainder had either moved out of the community or declined to participate in the study. This selection procedure was used to filter out people who mentioned rare or little-known species as this was counter to the goal of obtaining information regarding shared knowledge.

### Species selection

The 12 species mentioned most often during the previous interviews by the LEA team were selected (Table 2). In addition to their fruit, the cladode and tuber of *Pilosocereus pachycladus* subsp. *pernambucoensis* (Ritter) Zappi and *Spondias tuberosa* Arruda, respectively, were consumed, yielding a total of 14 edible items. Samples of most of the species were collected, taxonomically classified, and deposited at the UFP-Geraldo Mariz Herbarium at the Federal University of Pernambuco. Duplicates were also sent to the HUEFS herbarium of the State University of Feira de Santana.

**Table 2:** Native wild edible species targeted in the present study in NE Brazil

**Common name**	**Family**	**Species**	**Part(s) of the plant used as food**
Batinga	Myrtaceae	*Eugenia* sp.	Fruit
Cana de macaco	Marantaceae	*Maranta gibba* Sm	Stalk
Coco catolé	Arecaceae	*Syagrus cearensis* Noblick	Fruit
Facheiro	Cactaceae	*Pilosocereus pachycladus* subsp. *pernambucoensis* (F. Ritter) Zappi	Fruit and cladode
Imbu	Anacardiaceae	*Spondias tuberosa* Arruda	Fruit and tuber
Incó	Brassicaceae	*Neocalyptrocalyx longifolium* (Mart.) Cornejo & Iltis	Fruit
Jatobá	Fabaceae	*Hymenaea courbaril* L.	Fruit
Mandacaru	Cactaceae	*Cereus jamacaru* DC.	Fruit
Manofê	Apocynaceae	*Mandevilla tenuifolia* (J.C. Mikan) Woodson	Rhizome
Pirim	Myrtaceae	*Psidium schenckianum* Kiaersk.	Fruit
Trapiá	Brassicaceae	*Crateva tapia* L.	Fruit
Ubaia	Myrtaceae	*Eugenia pyriformis* Cambess.	Fruit

### Data collection

Informal conversations with 30 community members were conducted. The final semi-structured interviews took place between September 2009 and May 2010 [32,33]. The interviews each took between 50 minutes and 2,5 hours.

People were asked if they were familiar with each of the 14 edible items, their past and present uses, the good and bad characteristics associated with each (hereafter referred to as “positive and negative perceptions”), and the location where plants are collected. Additional questions regarding the interviewee’s socioeconomic characteristics were included (i.e., age, gender, current occupation, previous occupation, monthly family income, and monthly individual income). For the positive and negative perceptions, no categories were created prior to data collection, to avoid skewing the answers.

The participants were asked what they thought about eating *bush plants* (wild edible plants), emphasizing the reasons why they had abandoned their consumption. The answers to this open question were transcribed literally for collective subject discourse analysis.

### Data analysis

Answers to the semi-structured interviews were categorized to facilitate analysis, and percentages were calculated for each.

To evaluate the relationship between socioeconomic factors and plant perceptions, we conducted a simple linear regression analysis of normally distributed, continuous variables; Pearson and Spearman correlation analyses of normally and non-normally distributed data, respectively; and contingency table analysis for nominal variables. All analyses were performed using the BioEstat 5.0 software [34]. The analyses performed for each hypothesis are shown in Table 3.

**Table 3:** Statistical analyses used to evaluate hypotheses on the use of edible plants in a local community in NE Brazil

**Hypothesis**	**Variables**	**Statistical test used**
The most-consumed plants will be those that are perceived as tasting better.	Number of citations of current use of plant x vs. Number of citations of pleasant flavor of plant x	Pearson or Spearman correlation analysis
The most-consumed plants will be those that people perceive to be the most commercialized ones.	Number of citations of current use of plant x vs. Number of people who cited plant x as being marketable	Pearson or Spearman correlation analysis
Less-consumed plants will be those that are negatively perceived by the community.	Number of citations of current use of plant x vs. Number of citations of negative perception of plant x	Pearson or Spearman correlation analysis
Less-consumed plants will be those that are perceived as less abundant.	Number of citations of current use of plant x vs. Number of citations of low abundance of plant x	Pearson or Spearman correlation analysis
Less-consumed plants will be those that are perceived as less available.	Number of citations of current use of plant x vs. Number of citations of low availability of plant x	Pearson or Spearman correlation analysis
People with lower incomes will have more perceptions that encourage the use of wild edible plants.	Monthly family income	Simple linear regression analysis
	Monthly individual income vs. Number of positive perceptions	
People who perform agricultural activities in the community will have more perceptions that encourage the use of wild edible plants.	Current occupation	Contingency tables
	Past occupation vs. Ratio of positive/negative perceptions	
People with higher incomes will have more perceptions that limit the use of wild edible plants.	Monthly family income	Simple linear regression analysis
	Monthly individual income vs. Number of negative perceptions	

Through the collective subject discourse analysis technique [35], the discourse of participants concerning their opinion on use of wild plants as food was constructed.

### Ethical aspects

As mentioned above, this investigation was part of a larger project that had the consent of the community. Those interested in participating signed a Free and Informed Consent Form (TCLE, *Termo de Consentimento Livre e Esclarecido*) in accordance with resolution 466/2012 of the National Health Council. Permission to record the interviews was obtained from the participants, before they took place.

The project was approved by the Ethics Committee on Human Research from the Health Science Center of the Federal University of Pernambuco (entry number 238/06) [21,28,30,31,36].

## Results

### Socioeconomic characteristics of the interviewed population

The 39 participants were between 27 and 90 years old (mode = 71, mean = 58, SD = 16.38). Twenty-seven were women, and 12 were men. Ninety-two percent had once been farmers, 33% were currently farmers, 46% were retired, and 21% had other occupations. In terms of family income, 31% of participants earned less than minimum wage (US$ 229), 66% earned between one and two times the minimum wage, and the remaining 3% earned over twice the minimum wage. In terms of individual income, 39% earned less than minimum wage, 56% earned minimum wage, and 5% earned more than minimum wage.

### Past and present consumption of native wild edible plants

Of the 14 edible items, 7 were reported as known to all interviewed. Three (*S. cearensis*, *P. pachycladus* cladode and *S. tuberosa* fruit) were reported as consumed by all interviewees at some point in their lives. The most consumed item was the fruit of *S. tuberosa*, with 97% current consumption.

### Perceptions of native wild edible plants

The answers regarding the good and bad characteristics of each edible item were classified into 18 categories (Table 4). The most frequently cited positive characteristics; i.e., those that encouraged consumption, were the *organoleptic characteristics of the edible part* (52%; more specifically, the *flavor*, which comprised 48% of the total positive citations), *versatility* (24%) and *ways of preparing* (6%) (Table 4). The most cited negative characteristics were the *organoleptic characteristics of the edible part* (28%; more specifically *flavor*, which comprised 14% of the total negative citations), *harvest, consumption and processing* (14%), *availability* (13%), and *seasonality* (8%) (Table 4).

**Table 4:** Categories included in the data analysis on the use of edible plants in a local community in NE Brazil

**Category**	**Description**			
	**Positive aspects**	**Frequency**	**Negative aspects**	**Frequency**
**Availability**	Plants that are easy to find because they grow near the community.	28/612 (4.58%)	Participants mentioned difficulty in finding the plant in question, indicating that the species was not present near the community.	95/724 (13.12%)
**Biophilia and conservation**	People stressed the importance of a plant for the survival of other individuals in the ecosystem, for the conservation of the environment, for their beauty, or because they are considered safe and therefore have “*a right to survive*”.	23/612 (3.76%)	Interviewees stated that the use of a given plant and/or particular food item could threaten the plant’s conservation in the region.	1/724 (0.14%)
**Commercialization**	Plants that are currently, or potentially could be, commercialized.	5/612 (0.82%)	Plants that cannot be commercialized or sold.	4/724 (0.55%)
**Cultural acceptance**	Opinions expressed by participants who stressed the importance of a plant in preparing traditional food and/or because the foods are highly valued. Shared knowledge is considered culture in this study.	16/612 (2.61%)	Plants that are stigmatized, e.g., as food for poor people, food for children, or food that appearance is unpleasant. Shared knowledge is considered culture in this study.	54/724 (7.46%)
**Emergency food**	Includes opinions about plants used in times of scarcity.	4/612 (0.65%)	-	0/724 (0%)
**Harvest, consumption and processing**	-	0/612 (0%)	Participants mentioned some difficulty in collecting the plant because of thorns, associations with poisonous animals, difficult access to the edible part, or other factors that make consumption and processing difficult (e.g., abundance of seeds and difficulty in preparing).	103/724 (14.23%)
**Mitigating hunger and thirst**	Participants refer to plants with properties that satiate hunger or thirst.	24/612 (3.92%)	Participants refer to plants that cannot satiate hunger or thirst; plants that can be consumed but that cannot satiate.	12/724 (1.66%)
**Organoleptic characteristics of the edible part**	Pleasant flavor, pleasant smell, thin skin, appropriate size, appropriate amount of water, appropriate amount of pulp.	317/612 (51.8%)	Unpleasant flavor, unpleasant smell, thick skin, small size, among others.	199/724 (27.49%)
		Flavor 295/612 (48.2%)		Flavor 104/724 (14.4%)
		Smell 10/612 (1.63%)		Size 55/724 (7.6%)
		Size 9/612 (1.47%)		Smell 22/724 (3%)
		Amount of pulp 3/612 (0.49%)		Amount of pulp 11/724 (1.5%)
				Core size 7/724 (1%)
**Perception of health effects**	Participants reported the use of plants due to their healing properties or their ability to promote wellness.	4/612 (0.65%)	Indicate the avoidance of plants due to their damaging properties or because they produce unpleasant side effects (e.g., belly ache).	22/724 (3.04%)
**Phenotypic and ontogenetic diversity**	-	0/612 (0%)	Participants indicated that not all plants produce equally good edible parts, mainly when referring to their flavor.	48/724 (6.63%)
**Impossibility of eating when you are older**	Answers mentioning a positive aspect of the plant’s characteristics (sugar, fat content, pulp consistency), thus allowing its consumption by older people.	2/612 (0.33%)	Respondents mentioned that the plant could not be consumed by some people due to characteristics that preclude its consumption by older people (very hard edible parts, for which people need to have teeth to consume them).	11/724 (1.152%)
**Seasonality**	Opinions expressed by people who stressed the characteristics of some plants that have edible parts that are not very seasonal, which means that it is almost always easy to find the edible organs.	3/612 (0.49%)	Answers that were mentioned as limiting the consumption of some plants because many of them produce edible organs only during a specific and/or short time of the year (usually refers to fruits because the other edible organs are often not seasonal).	59/724 (8.15%)
**Texture**	Plants whose edible organs have pleasant textures that encourage their consumption.	3/612 (0.49%)	Responses mentioned as limiting factor the unpleasant texture.	39/724 (5.39%)
**Unaware of the resource**	-	0/612 (0%)	Participants stated that they did not know that the plant in question was used as food.	44/724 (6.08%)
**Versatility**	Includes plants that the participants described as having other services besides their use as food.	148/612 (24.18%)	Participants stated that the plant in question did not have other uses than being edible were considered negative.	9/724 ( 1.24%)
**Vulnerability to pests and susceptibility**	Answers that mentioned the capacity of the alimentary organ to resist plagues or adverse environmental conditions and remain in a good state/condition for a long time.	1/612 (0.16%)	Includes answers that mentioned that the edible organ of the plant is easily spoiled, which limits its consumption.	18/724 (2.49%)
**Ways of preparing**	Answers indicating that the plant in question has many different preparation methods.	34/612 (5.56%)	Answers indicating that the plant in question can be prepared in only a few ways.	4/724 (0.55%)
**Others**	-	0/612 (0%)	Answers given by a small number of people that could not fit into the other created categories, such as the fact that the plant could bring bad luck or that it could inhibit the growth of other plants around it.	2/724 (0.28%)

### Perceptions of native wild edible plants and their relationships to their use as food

The number of users of a given plant species increased with the number of positive reported perceptions (r (Pearson) = 0.82 for current consumption; r (Pearson) = 0.85 for past consumption; p < 0.01). In contrast, there was no relationship between the number of negative perceptions for each species and the number of current or past users.

The positive perceptions that had a significant correlation with current consumption were *pleasant flavor* (part of the *organoleptic characteristics* category) (Spearman r = 0.95; p < 0.01) and *highly valued food* (*cultural acceptance*) (Spearman r = 0.67; p < 0.01). The positive perception *emergency food* exhibited an inverse correlation with consumption (Spearman r = 0.67; p < 0.01). A positive relationship between consumption and availability was found, but it was significant only for past consumption (Spearman r = 0.46 for past consumption; p = 0.05; Spearman r = 0.53 for current consumption; p = 0.09). No significant correlations were found between the commercialization status of a plant and either its past or current consumption (p > 0.05).

Factors that limit the current consumption of native wild plants included a perceived association with poverty (*cultural acceptance*) (Spearman r = − 0.77; p < 0.01) and *unpleasant flavor* (part of the *organoleptic characteristics* category) (Spearman r = − 0.69 p < 0.01). The characteristic *small size* (part of the *organoleptic characteristics* category) was correlated with greater consumption (Spearman r = 0.60; p < 0.05). There were no significant relationships detected between availability and either past or current consumption (p > 0.05).

During the interviews and informal conversations, we observed that there were participants who placed higher value on those plants that could be prepared in a variety of ways, had several uses, and/or were used as medicine. Therefore, we tested the significance of these relationships. We found that only negative *cultural acceptance* was inversely correlated with the number of ways in which the plant could be prepared (Spearman r = −0.66; p < 0.01).

### Perceptions of native wild edible plants and their relationships with socioeconomic factors

Age was directly related to both negative (adjusted R^2^ = 18.18%; p < 0.05) and positive perceptions (adjusted R^2^ = 25.79%; p < 0.05). There was no correlation between perceptions and family income. In contrast, perceptions correlated with individual income, although only weakly (Spearman r = 0.33 with positive characteristics; Spearman r = 0.32 with negative characteristics; p < 0.05). There were no significant relationships between perceptions and genus, past occupation, or current occupation (p > 0.05).

### Collective subject discourse

Ten central ideas were identified that largely concurred with the 18 categories of the perception analysis. Due to the high number of central ideas and the extensive key expressions, the latter are not shown.

1. Central idea: Organoleptic characteristics of the edible part

“Those kinds of plants are good because they serve as food, they do not offend us. Sometimes we eat those things just like that, not because we are hungry, but because we think they are delicious and we eat them. Some of them are good, the sweet ones; sometimes they are really bad, sour, bitter.”

2. Central idea: Negative cultural acceptance

*“I don’t like eating plants from the woods. Not everybody tastes those plants from the woods, they are not fruits that are planted. [Those] plants are useless, plants without any importance. Now people eat them less because in the past there was more crisis. It was a bad time; in the old days there was no winter, no rain, no help from the government, there was nothing, and people needed them. [It’s been some years] when we were working and felt hungry, we would eat those plants and it was ok; [we would eat them] for hunger, need, not for the taste. One thing is* batata do chão*, that the person eats because they have nothing else to eat, [but] I wouldn’t go out to look for* ubaia*, or climb the mountain for* batinga *or* facheiro*. Wouldn’t go because I don’t need to and it’s not worth the trip. Nowadays after retirement people don’t get those things to eat anymore, they just want good things, have everything at home, but when I was a little girl, when I was younger, I would eat them, just for fun, but nowadays no one is starving anymore. When you are a child you don’t refuse a sweet fruit, no, you eat them all. Now the children don’t eat this kind of fruit, don’t even look at them, don’t have the desire to go where those things are because they have other things at home. It is fantasy because it is not useful for medicine, nor for anything.”*

3. Central idea: Opportunistic consumption of wild plants

“*Those little fruits we ate when we were working on the fields and got hungry, when I was coming home from the farm, I would eat one. You eat them because sometimes they appear there in the woods, then you get that little fruit. Before, we used to walk more by the mountains, where there are more of them. Now the people don’t walk so much in the woods as before, but long ago… If you are going by and there is some, it’s alright if I find it like this, I eat it.*”

4. Central idea: Positive cultural acceptance

*“If those fields were mine, I wouldn’t let them destroy everything because the people will be like “no, I have heard [of these plants], but I don’t know them”, it will be like that in the future of those young people. I think that [those plants] are good, thank God, because people can eat them when they are in need and without. If it is edible and up to now you haven’t discovered it, from the moment you discover that there is some in our region, I think you should really use it, take the opportunity. My kids know about those plants also, they are used to them. The same way my father taught me, together with my mom, I also tell them. Then they eat just like I do. The* facheiro*, which is a blessed fruit, is an old tradition and it continues until today. I eat the* manofê *so it’s not forgotten.”*

5. Central idea: Impossibility of eating when you are older

*“*Cana de macaco*, when I was coming back from the farm, I would eat it, but nowadays nobody eats it anymore, nobody has the teeth for that anymore. The time for those things is over. We are getting older, sometimes nature is not the same, age doesn’t allow you to walk by the mountains, our legs ache.”*

6. Central idea: Seasonality of wild species

“Those fruits from the woods, we see them only from time to time, they only come when it is the rainy season. Many take time to come, but the plants should be here every day, not only at their time.”

7. Central idea: Decrease in resource abundance (availability) over time

“Before there were more, then it would produce more, but they took many of the trees that existed. There are almost no trees anymore. The people didn’t even notice that it is over.”

8. Central idea: Lost habit, lost knowledge

*“The tradition is about to end,* facheiro *candy, cassava and corn cake. The young people do not know them and do not value them. Now people know less because they are not very interested, then we forget about those things way back then. I used to eat more of those plants before, now there are some that I still eat a lot, and there are many that I used to eat more of before. Some 20 years from now, or maybe 30, nobody will look for these things. Today, the children don’t walk in the woods anymore; previously the parents would take their kids to work, and when they were hungry they would eat them, but not anymore. The children eat fewer plants from the woods than I used to eat before. In the younger group, they aren’t familiar with many of those things or know how to eat them, they think it is bad to eat those plants, they want to buy everything ready-made. I don’t see today a child doing what Edgar used to do and what we used to do, and it is learning from the world and learning from us. If I take one of those fruits and give it to a child, they may think that it is disgusting and won’t even want to taste it.”*

9. Central idea: Perception of health effects

*“I think it is good to eat those plants. They have some things that people need, we notice they are healthier, certainly they have something we don’t even know, vitamins, less sugar, less cholesterol, less damaging things. Each one has their kind of protein, and we need protein. Now the people eat* cana de macaco *because it is medicinal,* jatobá*,* juá*. This* cana de macaco *is good for cholesterol. When you eat two or three* ubaias, *your sickness stops. The* pirim *is good for your throat, for a bunch of things. Then I think that if you eat them they won’t harm you.”*

“I’m afraid of eating things that I don’t know, in case they cause illness. The people don’t like that kind of thing, you have to prepare, it can harm you.”

10. Central idea: Mitigating hunger and thirst

“It is good because it serves to stave off hunger; hunger is related to the devil. All those fruits from the woods we eat for sport, not because it satisfies. With weak food, we can hang on for two hours.”

## Discussion

Consumption of native wild edible plants in the Carão community is currently very limited and has decreased markedly, relative to the past levels.

Ten of the positive perceptions reported in this study are the same as those jointly reported by Ladio [11] and Grosskinski and Gullick [12] (*organoleptic characteristics of the edible part* (*flavor*), *versatility*, *availability*, *mitigating hunger and thirst*, *biophilia and conservation*, *cultural acceptance*, *commercialization*, *emergency food*, *perception of health effects*, *seasonality*). The remaining 8 categories classified in this study are not mentioned in previous research. In contrast, several of the perceptions described in the previous studies were not represented in the reports of the people interviewed in the present study. Examples include the sprouting of wild plants before cultivated ones (reported by Ladio [11]), the low cost of wild plants, the ability of wild plants to easily adapt to the environment, the absence of pesticides or chemicals in the maintenance of wild plants, and the ability to store wild plants for a long time without spoiling (reported by Grosskinsky and Gullick [12]).

Regarding negative perceptions, Ladio [11] and Prasad [37] do not report *flavor* among the reasons for not using a plant as food. However, this perception is mentioned by Grosskinski and Gullick [12]. In contrast, *cultural acceptance* and *species availability* were important limiting factors on consumption and were described in all three previous studies. In particular, *difficulty in harvesting, consuming and processing*; and *seasonality* were reported by Ladio [11] and Grosskinski and Gullick [12] but not by Prasad [37]. The categories *perception of health effects*, *mitigating hunger and thirst*, and *emergency food* were mentioned by Grosskinski and Gullick [12] but not by Ladio [11] or Prasad [37].

Negative perceptions that were not represented by the residents of Carão have also been reported in other studies. Examples include the need to ask landowners for permission to collect wild plants on their land (reported by Ladio [11]), the low productivity of wild edible plants, the low quality of wild plants, and the relationship between the consumption of wild plants and children’s diseases (reported by Grosskinski and Gullick [12]).

The positive perception that we found to be most strongly associated with consumption was *flavor* (p < 0.01), comparable with similar findings by other authors [11-17,38]. Cruz-García [13] suggested that changes in the valuation of flavor occur due to the introduction of new foods into the community by people with higher incomes (usually coming from the city) and the interactions of young people with modern culture. From this perspective, flavor may be considered an important variable.

The negative perception that most strongly limits the consumption of wild plants is *cultural acceptance* (p < 0.01). This perception is understood as the association of wild plant consumption with low social status. Similar observations have been reported previously [12,13,39-43].

Similarly, the perception *emergency food* was related to limited consumption. Plants used during periods of shortage in agricultural production, such as during the dry period when no economic assistance from the government is provided to enable residents to buy food, are currently being neglected.

We suggest that the decreased consumption of wild edible plants is related to changes in cultural acceptance, together with increases in available income and the introduction of new food resources into the community, in accordance with Cruz-García [13].

The association between the consumption of wild edible plants and the belief that it is “food for poor people” suggests that the concept of poverty should be reviewed along with its indicators, which were developed to measure the lack of material goods [44]. The development model promoted in these regions favors production for commercial purposes and the purchase of products with added value. This leads to the abandonment of resources that the community traditionally used to meet their dietary needs. The loss of such resources increases the population dependence on paid employment sources and government grants and reduces their options for ensuring food sovereignty.

We observed no correlation between the number of uses of a plant and its acceptance; however such a correlation was reported by Ekué and collaborators in a population in Benin [45]. Contrary to our expectations [13], we found no relationship between *cultural acceptance* and medicinal use; similar results were found by Rivera et al. [46]. We found that participants did not distinguish between plants for food use and medical use for those plants where the method of administration was the same for both purposes, also noted by Etkin and Ross [47].

Both positive and negative perceptions were correlated with participant age, supporting the hypothesis that the more contact people have with the plants, the more knowledge they will have about them [48] and, consequently, the more positive and negative perceptions they will hold.

Based on the collective subject discourse analysis, a tendency was identified in the central ideas about cultural acceptance and loss of knowledge (central ideas 2 and 8) towards the loss of traditional knowledge in younger generations, as reported in other regions [13,49-51]. This loss may reflect a lack of contact with and use of the natural environment, with the deterioration of local ecosystems and reduced availability of wild plants limiting contact between these resources and youths.

We found a relationship between edible plant perceptions and individual income. This finding is consistent with the central idea that wild plants are used primarily when other food is lacking (central idea 2). This pattern of use may occur because many of these perceptions related to edible plants are more of an individual than a family nature. Family income was difficult to estimate for many interviewees because they often did not know the amount of money that other family members contributed to the family budget. It is also likely that individual income is masking the age effect.

The results found here support the trends established by other studies conducted around the world, although we acknowledge that our work is focused in a local context and has limited generalizability. The aim of this study was to highlight the importance of studying perceptions in addition to knowledge and use. We used an indirect method of identifying the ideas regarding wild plants and their use, instead of directly asking for the reasons why a certain plant is - or is not - consumed. This was done in an effort to avoid the rationalization and filtration of answers from respondents, which could inhibit the accurate identification of the factors determining consumption.

We set out to test the proposed hypotheses, although the scope of the study goes beyond these objectives. This approach enabled us to obtain a general overview of the situation and identify other unexpected variables and relationships. This study was exploratory and suggests new variables for future studies.

## Final considerations

A set of related perceptions regarding the use of wild edible plants was identified in a rural *Caatinga* community. Although only *cultural acceptance*, *flavor*, and *emergency food* were significantly associated with consumption, other identified perceptions may suggest avenues for further studies. While we recognize the local nature of our findings, many of them were consistent with those from other regions of the world, suggesting that these results may reflect broader patterns.

This study identified a loss of knowledge in the younger generations, suggesting a negative impact of the development model on the conservation of knowledge and the use of traditional food resources. The arrival of a new model of development is accompanied by the stigmatization of traditional resources and their use, leading to their abandonment. Even for an apparently objective variable such as flavor, cultural transformation mechanisms can influence it.

The availability of edible wild plants in the *Caatinga* has been important in the past in tolerating food shortages during droughts. Currently, government grants have replaced this function. The result, in terms of food security and dependence on external aid for the population, needs to be studied.

The combined use of qualitative and quantitative methods allows a better comprehension of the results. The identified relationships suggest that changing perceptions may affect the conservation of plants, traditional practices and the associated knowledge.

## Competing interests

The authors declare that they have no competing interests.

## Authors’ contributions

MPC was responsible for field research and interviews. MPC and UPA designed the study. MPC and PMM processed the data and performed the quantitative analysis. All authors contributed to the manuscript. All authors have read and approved of the final manuscript.
